# Institutional modified post esophagectomy care protocol offers improved perioperative outcomes – a single-center propensity score matched analysis

**DOI:** 10.1186/s12957-026-04588-w

**Published:** 2026-10-03

**Authors:** H. C. Hillebrecht, A. Bührle, J. Wang, J. Hoeppner, M. K. Diener, S. Fichtner-Feigl, J. Hipp, J. Kuvendjiska

**Affiliations:** 1 Department of General and Visceral Surgery, Faculty of Medicine, Medical Center -University of Freiburg, University of Freiburg, Hugstetter Str. 55, Freiburg, 79106 Germany; 2 Department of Surgery , Bielefeld University, Medical School and University Medical Center OWL, Campus Hospital Lippe, Röntgenstr. 18, Detmold, 32756 Germany; 3 Department of Surgery, Paracelsus Medical University Nuremberg, Prof.-Ernst-Nathan-Str. 1, Nürnberg, 90419 Germany

**Keywords:** Esophagogastric junction cancer, Esophageal cancer, Ivor Lewis esophagectomy, Visceral surgery, Anastomotic leakage, Oral decontamination, Hydroxyethyl starch

## Abstract

**Background:**

Two-field esophagectomy for esophageal cancer and tumors of the esophagogastric junction is associated with substantial postoperative morbidity and mortality. Enhanced Recovery After Surgery (ERAS^®^) protocols aim to improve perioperative outcomes through standardized multimodal care pathways. At our institution, a modified perioperative regimen has been implemented that deviates from current ERAS^®^ recommendations, incorporating selective oral decontamination, an enforced near-zero fluid balance, colloid administration using hydroxyethyl starch (HES), and routine nasogastric decompression.

**Methods:**

We performed a retrospective analysis of 325 consecutive patients who underwent two-field esophagectomy. Of these, 279 were treated under the modified perioperative regimen, while 46 received standard care. To adjust for baseline differences, we conducted 1:1 propensity-score matching. Postoperative outcomes, including complication rates, anastomotic leakage, Comprehensive Complication Index (CCI) and textbook outcome rates were compared between groups.

This trial was prospectively registered (DRKS00024369) and approved by the Medical Ethics Committee of the University of Freiburg (Reg.-Nr. 21-1093 and 21-1713).

**Results:**

Prior to PSM, modified care group demonstrated a higher frequency of textbook outcomes and a significantly lower incidence of anastomotic leakage compared with standard care. Overall rates of major complications did not differ significantly between groups, though a trend toward reduced complication severity as measured by CCI was observed. After PSM, baseline characteristics were balanced. Rates of textbook outcome (Clavien-Dindo 0: 15.2% vs 28.3%, p=0.14) and severe complications (Clavien-Dindo ≥III: 39.1% vs 58.7%, p=0.095) were reduced but did not reach statistical significance. The CCI remained numerically lower in modified care (32.01 vs 35.70, p=0.061). Odds ratios for major complications (OR 0.45, 95% CI 0.20–1.04), anastomotic leakage (OR 0.30, 95% CI 0.06–1.59) and IMC readmission (OR 0.33, 95% CI 0.08-1.34) favored modified care but were not statistically significant. Fluid balance on POD4 and early parenteral nutrition were similar after matching. Acute kidney injury did not occur more frequently after HES-application. Overall and disease-free survival did not differ between groups.

**Conclusions:**

The modified perioperative regimen applied in this study appears to be a safe alternative to conventional ERAS®-based pathways. While definitive superiority could not be demonstrated after propensity matching, the observed trends — particularly regarding anastomotic integrity and overall complication burden — support further prospective evaluation of this approach.

## Introduction

Esophageal cancers (EC) and cancers of the esophagogastric junction (EGC) are among the most common malignancies worldwide [1, 2]. In the absence of distant metastases [3], esophagectomy remains the cornerstone of curative treatment for resectable disease.

Despite improvements in surgical technique, overall complication rates after esophagectomy often exceed 50% even at high-volume centers – most frequently pulmonary complications, anastomotic leakage, and atrial arrhythmias [4–6].

Postoperative morbidity is associated with increased risk of mortality and reduced oncological survival [7–9], decreases health-related quality of life [10, 11] and increases healthcare cost [12–14]. This justifies the need for multifactorial formats such as fast-track or “enhanced recovery after surgery” (ERAS^®^)-schemes to accelerate and improve convalescence after surgery. For esophageal resections, such a standard is largely based on extrapolated evidence and not yet widely adopted [15].

At our center, retrospective, translational and extrapolated evidence has led to the empirical adoption of a multifactorial post-esophagectomy modified care (MC) routine deviating from recent ERAS^®^ society recommendations.

Its key components oral decontamination (OD), enforced near-zero fluid balance, application of hydroxyethyl starch solution (HES) for fluid resuscitation, consequent nasogastric decompression with liquid diet for the first four postoperative days and early parenteral nutritional support are summarized in Table 1.

**Table 1:** Comparison of institutional standard of care, modified care and ERAS^®^-society recommendations

	Routine care	Modified care	ERAS^®^ (15).
Oral decontamination:	none	250 mg Vancomycin *p*.o. 1-1-1-1 500 mg Paromomycin *p*.o. 1-1-1-1 100 mg Amphotericin B *p*.o. 1-1-1-1	“Routine prolonged postoperative antibiotic treatment is not supported; instead, appropriate perioperative prophylaxis should be applied.”
Fluid balance		Maximum positive balance of </= 4 kg up to POD 4	“Optimal fluid balance should be the focus with consideration of all contributory factors, positive balance resulting in weight gain > 2 kg/day is to be avoided.”
Solution for fluid resuscitation	Balanced crystalloids	Routine application of 500 ml/dHydroxyethyl starch (HES) to POD 4	“Balanced crystalloids use for fluid replacement is recommended.”
Diet, early nutritional support	Introduction of enteral nutrition as tolerated.	Strict liquid diet nasogastric decompression tube until POD 4	“Introduction of early enteral nutrition is beneficial in patients undergoing surgery for esophageal cancer”
Early nutritional support		Parenteral nutrition	“oral or jejunostomy”

Selective oral decontamination has been observed in some trials to achieve a trend towards reduced rates of anastomotic leakage and pneumonia after gastrointestinal cancer surgery other than esophagectomy [16, 17].

Fluid overload represents an independent risk factor for esophagogastric anastomotic leakage and postoperative complications [18–28], ERAS^®^-society guidelines recommend avoiding “positive balance >2kg/day”. Our modified regimen calls for even more restrictive management and generous use of diuretics [18] to not exceed positive balance of >/= 4 kg up to POD 4. In hypotensive patients, a combination of clinical, sonographic and laboratory parameters was used to assess the need for fluid resuscitation, vasoplegia in absence of hypovolemia was instead treated with vasopressors.

Anesthesiological as well as ERAS^®^-society recommendations rely on balanced crystalloids as standard for fluid resuscitation – especially since an influential trial [29] demonstrated an increased need for renal replacement therapy and blood transfusion after application of HES in critically ill patients. Our modified regimen routinely employs HES for its potential benefits in achieving the desired near-zero balance as demonstrated in animal models [27, 30].

In contrast to guidelines recommending no-tube, no-fasting regimens [15, 31, 32], we consider routine nasogastric decompression and consequent liquid diet necessary for the immediate postoperative course. The high prevalence of postoperative delayed gastric conduit emptying in combination with early oral nutrition is considered to be a cause of significant morbidity and mortality due to e.g. silent or frank aspirations. We consider placement of nasojejunal tubes or catheter-jejunostomies for nutritional support overtreatment for most patients who are usually able to tolerate oral intake by postoperative day 4–5. And instead rely on parenteral nutrition for early nutritional support in the modified regimen contrary to society recommendations favoring enteral routes.

It was the aim of this study to describe our single, high-volume center experience and evaluate safety and efficacy of the distinct institutional regimen summarized in Table 1 deviating from ERAS^®^-society recommendations.

## Methods

This cohort study is reported in accordance with the STROBE-statement [33]. Patient-, disease and surgery-specific data of patients undergoing esophagectomy for cancer 01/2014 to 01/2021 were extracted from patient records, collected in our database, and retrospectively analyzed (DRKS00024369). The Medical Ethics Committee of the University of Freiburg approved the study (Reg.-Nr. 21-1093 and 21-1713).

Included were all consecutive patients undergoing Ivor Lewis-esophagectomy for cancer. Staging was performed using available chest and abdomen computed tomography and endoscopy with endoscopic ultrasound. Tumor localization and clinical stage were defined according to the 8th edition of the UICC staging system [34].

During the observed period, standard approach was hybrid minimally invasive esophagectomy combining laparoscopy and thoracotomy, no conversion laparotomy was necessary. Standard reconstruction consisted of circular stapled end-to-side-esophagogastrostomy. Pyloroplasty, pyloric drainage or dilatation was not routinely performed. Operating surgeons did not differ significantly between groups (neither overall nor matched cohort), all expert esophageal surgeons with completed learning curve before the trial period. Patients routinely received thoracic epidural anesthesia (TEA) and non-opioid analgesia (standard Metamizole 1000 mg 4x/d) in both cohorts with parenteral opioids (mainly transdermal fentanyl) as fallback only when TEA was unsuccessful. All patients were routinely admitted to IMC for the first four postoperative days. In MC, a combination of clinical, sonographic and laboratory parameters was used to assess the need for additional fluid resuscitation, vasoplegia in absence of hypovolemia was instead treated with vasopressors. No specific target was set for early mobilization after esophagectomy. We generally aim to mobilize out of bed early (minimum 15 min bedside sitting on POD 0 with daily increases to at least 2 × 2 h per day bedside sitting and ambulation if tolerated), but this is not specific to post-esophagectomy care (or either study group).

The primary hypothesis was that a modified care (MC) regimen as summarized in Table 1 would be associated with reduced morbidity when compared to a standard of care (SC). We considered treatment adhering to all measures as modified care, treatment without any of the interventional measures was considered routine care.

A reduction of Comprehensive Complication Index (CCI) was chosen as the primary endpoint because it provides a more comprehensive and sensitive assessment of postoperative morbidity than dichotomous or single-complication endpoints. Treating the CCI as a continuous variable preserves information and enhances statistical power, which is particularly relevant in retrospective analyses. As this was a retrospective study with a fixed sample size, a post hoc detectable effect size calculation was performed for the primary endpoint rather than a prospective sample size calculation. Given the retrospective design and fixed cohort size, a post hoc detectable effect size calculation showed that the study was powered to detect a minimum difference of approximately 6.7 points in mean Comprehensive Complication Index between groups (two-sided α = 0.05, power = 80%). All other endpoints were analyzed in an exploratory manner and should therefore be interpreted as hypothesis-generating rather than confirmatory. Routine contrast swallow was performed on POD 4 in all patients to screen for anastomotic leakage. In addition, clinical suspicion of anastomotic leakage was raised by fever, clinical signs of inflammation or sepsis, unexplained pulmonary or cardiac complications, or laboratory abnormalities such as leukocytosis or elevated C-reactive protein. In cases of clinical suspicion, CT with oral contrast was performed. Anastomotic leakage was confirmed endoscopically in all cases classified as anastomotic leakage. This surveillance and diagnostic approach was applied consistently throughout the study period and in both treatment groups.

### Data extraction

The following data were extracted from our prospectively maintained database: *Demographic and patient specific* data: Age, Gender, Body-Mass-Index (BMI), American Society of Anesthesiology physical status class (ASA score), RCS Charlson-Comorbidity-Index [35], information on cardiac, vascular, pulmonary, renal comorbidity;

#### Disease-specific data

 Histological Subtype of EC, Pretherapeutic TNM-Stage, neoadjuvant therapy information; pretherapeutic and pathological staging;

#### Surgical data

surgical procedure and perioperative data including overall complications according to the comprehensive complication index (CCI) [36];

#### Postoperative care data 

diuretic use, fluid balance, serum creatinine levels at baseline and over the first four postoperative days, length of stay in IMC/hospital, readmission rates to IMC/hospital;

#### Histopathological data

postneoadjuvant TNM-Stage including residual tumor-classification (R-Status) [37];

#### Oncological data

Type of (neo-)adjuvant treatment, time to adjuvant treatment, completion rate for adjuvant treatment, overall and disease free survival.

### Statistical analysis

Survival data were systematically obtained from the cancer registry of our Comprehensive Cancer Center (CCCF) at the University Hospital of Freiburg. Follow-up-data were collected until February 2022. Results are expressed as mean (95% confidence interval, 95CI) or as number (per cent). Statistical analysis was performed using SPSS Version 28.0.1.0 (IBM Corp., Armonk, New York, USA) and R version 4.4.2 (R Foundation for Statistical Computing, Vienna, Austria) with R-Studio version 2024.09.1 + 394 (R Studio Inc., Boston, MA, USA) and additional packages CBCgrps, tableone, lmtest, matchit. Differences were considered statistically significant for *p* < 0,05.

We used Mann-Whitney-U-test for descriptive analysis of non-parametric variables and Pearsons’s chi-squared-test for categorical variables.

For propensity score matching using the *matchit* R package (v4.6.0), an optimal pair matching method was used after estimation of propensity scores using a generalized linear model. Propensity score differences served as distance metric and were estimated through logistic regression. To enhance matching quality and mitigate potential bias, a caliper was applied to ensure matched units would not exceed a specified threshold of propensity score difference. The caliper was set at 0.1 times the standard deviation of the propensity score. PSM was employed to match for baseline measures (see Table 1). Out of the original 325 samples, 92 patients were therefore included in 1:1 matching.

After PSM, odds ratios with 95% confidence intervals were estimated for binary outcomes. In outcomes with sparse data or complete/quasi-complete separation (zero cells), Firth penalized logistic regression was used; otherwise standard logistic regression was applied.

## Results

All consecutive patients with esophageal cancer and adenocarcinoma of the gastroesophageal junction (GEJ) undergoing Ivor Lewis-esophagectomy were included in this study and stratified according to postoperative treatment routine. Details comparing baseline characteristics between cohorts are shown in Table 2. From our database of patients with upper gastrointestinal cancers during that period, this yielded 325 unique entries of esophageal malignancies, 65 cases (20%) of squamous cell carcinomas (SCC) and 260 cases (80%) of adenocarcinomas. Most adenocarcinomas were GEJ-type tumors (Siewert I: 119 cases, 36,7%; Siewert II: 111, 34,3%; Siewert III: 17, 5,2%) with only few esophageal adenocarcinomas (12 cases, 3,7%). Histological subtypes and grading were not significantly different between groups.

**Table 2:** Baseline characteristics between unmatched and matched cohort

			unmatched (*n* = 325)							matched			
			SC (*n* = 46)			MC (*n* = 279)				MC (*n* = 46)			p
			n	%	median (IQR)	n	%	median (IQR)	p	n	%	median (IQR)	
Sex	female		13	28.3%		51	18.3%		0.16	13	28.3%		1
Age [y]					65.50 (60.00–72.75)			64.00 (58.50–73.00)	0.521			64.00 (59.00–71.75)	0.891
BMI [kg/m2]					25.10 (24.28–26.03)			25.65 (25.21–26.22)	0.95			25.74 (24.93–28.63)	0.625
ASA Score	ASA 1		0	0.0%		0	0.0%		0.093	0	0.0%		0.403
	ASA 2		17	37.0%		107	38.4%			15	32.6%		
	ASA 3		27	58.7%		161	57.7%			27	58.7%		
	>/= ASA 4		2	4.3%		11	3.9%			4	8.7%		
Comorbidities	cardiac		24	61.5%		157	65.1%		0.797	22	56.4%		0.818
	vascular		0	0.0%		17	6.1%		0.173	7	15.2%		0.018
	pulmonary		14	30.4%		48	17.2%		0.034	14	30.4%		1
	renal		3	6.5%		13	4.7%		0.863	2	4.3%		1
	diabetes		5	10.9%		33	11.8%		1	5	10.9%		1
RCS Charlson comorbidity index	0		0	0.0%		0	0.0%		0.776	0	0.0%		0.822
	1		21	45.7%		144	52.2%			19	41.3%		
	2		14	30.4%		80	29.0%			13	28.3%		
	>/=3		11	23.9%		52	18.8%			14	30.4%		
histological subtype	SCC		12	26.1%		53	19.1%		0.066	10	21.7%		0.55
	AC	non-GEJ	3	6.5%		9	3.2%			1	2.2%		
		Siewert I	12	26.1%		107	38.5%			9	19.6%		
		Siewert II	17	37.0%		94	33.8%			22	47.8%		
		Siewert III	2	4.3%		15	5.4%			4	8.7%		
Clinical T stage	</=T2		16	37.2%		78	30.6%		0.525	17	41.5%		0.215
	T3		25	58.1%		160	62.7%			22	53.7%		
	T4		2	4.7%		17	6.7%			2	4.9%		
Clinical N stage	cN+		26	63.4%		184	73.6%		0.246	31	75.6%		0.159
Clinical M stage	cM1		5	11.1%		20	7.4%		0.585	1	2.3%		0.215
pretherapeutic Grading	G0-1		18	39.1%		17	36.9%		0.972	13	28.3%		0.339
	G2		17	37.0%		19	41.3%			18	39.1%		
	G3		11	23.9%		10	21.7%			15	32.6%		
Neoadjuvant Chemotherapy	yes		23	50.0%		149	53.4%		0.788	21	45.7%		0.835
		FLO/(T)	23	50.0%		138	49.5%		0.89	22	47.8%		0.588
		other	0	0.0%		11	3.9%			3	6.5%		
Neoadjuvant Chemoradiation	yes		14	30.4%		89	31.9%		0.979	14	30.4%		1
		CROSS	11	23.9%		80	29.7%		0.137	14	30.4		0.503
		other	3	6.5%		9	3.3%			0	0%		
laparoscopic approach			36	78.3%		216	77.4%		0.846	34	73.9%		0.807
OP duration [min]					306.50 (296.00–318.00)			337.00 (296.50–383.00)	*p* = 0,01			316.00 (272.75–347.50)	0.851
intraoperative transfusion			2	4.3%		14	5.0%		1	3	6.5%		1

Most cases were locally advanced at diagnosis: 185 (57%) and 19 (6%) patients presented with cT3 or cT4 stages respectively. Overall, 65% of patients had lymph node involvement at diagnosis, therefore, most patients (85%) received neoadjuvant treatment, most frequently chemotherapy (FLO(T)), chemoradiotherapy (CROSS) was less common. Frequency and type of pretreatment did not differ significantly between groups.

Comorbidities, as reflected through ASA scores, RCS Charlson comorbidity index and frequency of known relevant comorbidities were evenly distributed between groups. It is of note, however, that in the unmatched cohort, more patients from the standard care group had concomitant pulmonary comorbidities (30% vs. 17%, *p* = 0,034) and operating time was significantly longer in the MC group (337 min vs. 307 min, *p* = 0,01).

We conducted propensity score matching to adjust for preoperative patient characteristics. After PSM, preoperative and demographic characteristics were evenly matched between groups, no significant differences remained (Table 2).

A comparison of postoperative morbidity as well as treatment related measures was performed in the overall as well as the matched cohort (Tables 3 and 4). In the unmatched cohort (*n* = 325), textbook postoperative courses (Clavien-Dindo 0) were significantly more frequent in the modified-care group compared with standard care (31.2% vs. 15.2%, *p* = 0.026), while severe complications (Clavien-Dindo ≥ 3) occurred less often (39.1% vs. 58.7%, *p* = 0.019), this included endoscopic pyloric dilatations in ~ 24% of unmatched cases (n. s. between groups before or after PSM). The overall burden of postoperative morbidity, reflected by the Comprehensive Complication Index (CCI), was also significantly lower in the modified-care group (median 28.92 [25.6–32.23] vs. 35.70 [29.60–44.90], *p* = 0.012). Among specific complications, overall frequency of anastomotic leakage was remarkably low and further and significantly reduced in MC (3.2% vs. 13.0%, *p* = 0.003) (Table 4).

**Table 3:** Comparison of treatment related measures between unmatched and matched cohort

	unmatched (n=325)							matched			
	SC (*n* = 46)			MC (*n* = 279)				MC (*n* = 46)			
	*n*	%	median (95CI)	*n*	%	median (95CI)	*p*	*n*	%	median (95CI)	*p*
loop diuretics used	38	82.6%		238	85.3%		0.802	40	87%		0.772
fluid balance on POD 4			1036.50 (-644.75–2531.50)			774.00 (-595.00–2253.50)	0.038			584.00 (-86.00–1181.00)	0.098
early postoperative parenteral nutrition (before POD5)	4	8.7%		92	33.0%		<0.01	10	21.7%		0.04
duration parenteral nutrition [days]			1.1 (0.8 - 2.06)			2.54 (1.96 - 3.13)	0.044			2.6 (0.9 - 2.9)	.184
nasogastric tube [days]			6.00 (5.00–6.00)			6.00 (5.00–6.00)	1			6.00 (5.00–6.00)	0.688

**Table 4:** Comparison of postoperative morbidity between unmatched and matched cohort

	unmatched (*n* = 325)							matched			
	SC (*n* = 46)			MC (*n* = 279)				MC (*n* = 46)			
	*n*	%	median (IQR)	*n*	%	median (IQR)	*p*	*n*	%	median (IQR)	*p*
Clavien-Dindo 0	7	15.2%		87	31.2%		0.026	13	28.3%		0.14
Clavien-Dindo >/=3	27	58.7%		109	39.1%		0.019	18	39.1%		0.095
30 day mortality	2	4.3%		8	2.9%		0.938	1	2.2%		1
CCI			35.70 (21.32–51.60)			28.92 (25.6–32.23)	0.012			32.01 (23.56–40.45)	0.061
Anastomotic leakage	6	13.0%		9	3.2%		0.003	2	4.3		0.267
Delayed gastric emptying	0	0.0%		20	7.2%		0.122	3	6.7%		0.233
Wound infection	2	4.3%		6	2.2%		0.706	1	2.2%		1
respiratory complication (pneumonia, insufficiency, reintubation, ARDS)	1	2.2%		7	2.5%		1	2	4.3%		1
acute kidney injury	4	8.7%		11	3.9%		0.296	4	8.7%		1
need for renal replacement therapy	0	0.0%		0	0.0%		1	0	0.0%		1
length of IMC stay [days]			6.00 (5.00–10.75)			6.00 (6.00–8.00)	0.255			6.00 (6.00–8.75)	0.698
IMC/ICU readmission	8	17.4%		24	8.6%		0.115	3	6.5%		0.199
Length of stay [days]			17.50 (14.00–24.00)			15.00 (12.50–21.50)	0.087				0.641
Weeks to adjuvant treatment [weeks]			7.00 (6.00–10.00)			7.00 (6.00–9.00)	0.909			7.00 (6.00–9.00)	0.412

Acute kidney injury (increases in serum creatinine as defined in KDIGO definition [38]) was uncommon and did not differ significantly between groups (3.9% vs. 8.7%, *p* = 0.296). Renal replacement therapy was not required in either group. Respiratory complications were rare and comparable (2.5% vs. 2.2%, *p* = 1), and surgical site infections, for which a potential benefit of selective decontamination has been postulated, were not significantly reduced (2.2% vs. 4.3%, *p* = 0.706).

The length of IMC stay durations were similar between groups (median 6.0 [6.0–8.0] vs. 6.0 [5.0–8.75] days, *p* = 0.255). IMC/ICU readmissions and overall hospital length of stay were numerically less and shorter in modified care but did not reach statistical significance (8.6% vs. 17.4%, *p* = 0.115 and 15.0 [12.5–21.5] vs. 17.5 [14.0–24.0] days, *p* = 0.087, respectively). Delayed gastric conduit emptying – defined as repeated vomiting requiring reinsertion of a nasogastric tube [51] – occurred more frequently in the modified-care group (7.2% vs. 0%), although this difference was not statistically significant (*p* = 0.122). The timing to adjuvant therapy was similar between groups (7.0 [6.0–9.0] vs. 7.0 [6.0–10.0] weeks, *p* = 0.909).

Albeit use of loop diuretics was comparable between groups (85.3% vs. 82.6%, *p* = 0.802), fluid balance on postoperative day 4 was significantly lower in the modified-care group (median 774 ml vs. 1036.5 ml, *p* = 0.038). Early postoperative parenteral nutrition (before POD5) was employed more frequently in modified care (33.0% vs. 8.7%, *p* < 0.01) and remained more common after PSM but did not reach statistical significance (21.7%, *p* = 0.147). Duration of parenteral nutrition was longer in the modified-care group in the unmatched cohort (2.54 vs. 1.1 days, *p* = 0.044), but this difference was no longer significant after matching (*p* = 0.184). Duration of nasogastric tube placement was identical between groups (median 6 days; *p* = 1 unmatched, *p* = 0.688 matched).

After propensity score matching (1:1; SC *n* = 46 vs. MC *n* = 46), differences in global complication metrics were attenuated and no longer statistically significant. Rates of Clavien-Dindo 0 (28.3%, *p* = 0.14) and Clavien-Dindo ≥ 3 (39.1%, *p* = 0.095) in the matched modified-care group did not differ significantly from standard care. The CCI remained numerically lower in matched modified care but remained shy of statistical significance (32.01 [23.56–40.45] vs. 35.70 [21.32–51.60], *p* = 0.061). Individual postoperative complications like respiratory complications, acute kidney injury, surgical site infection, and IMC readmission, likewise did not differ significantly after matching. Anastomotic leakage (4.3% vs. 13.0%, *p* = 0.26) remained reduced in MC but not statistically significant.

**Fig. 1:**
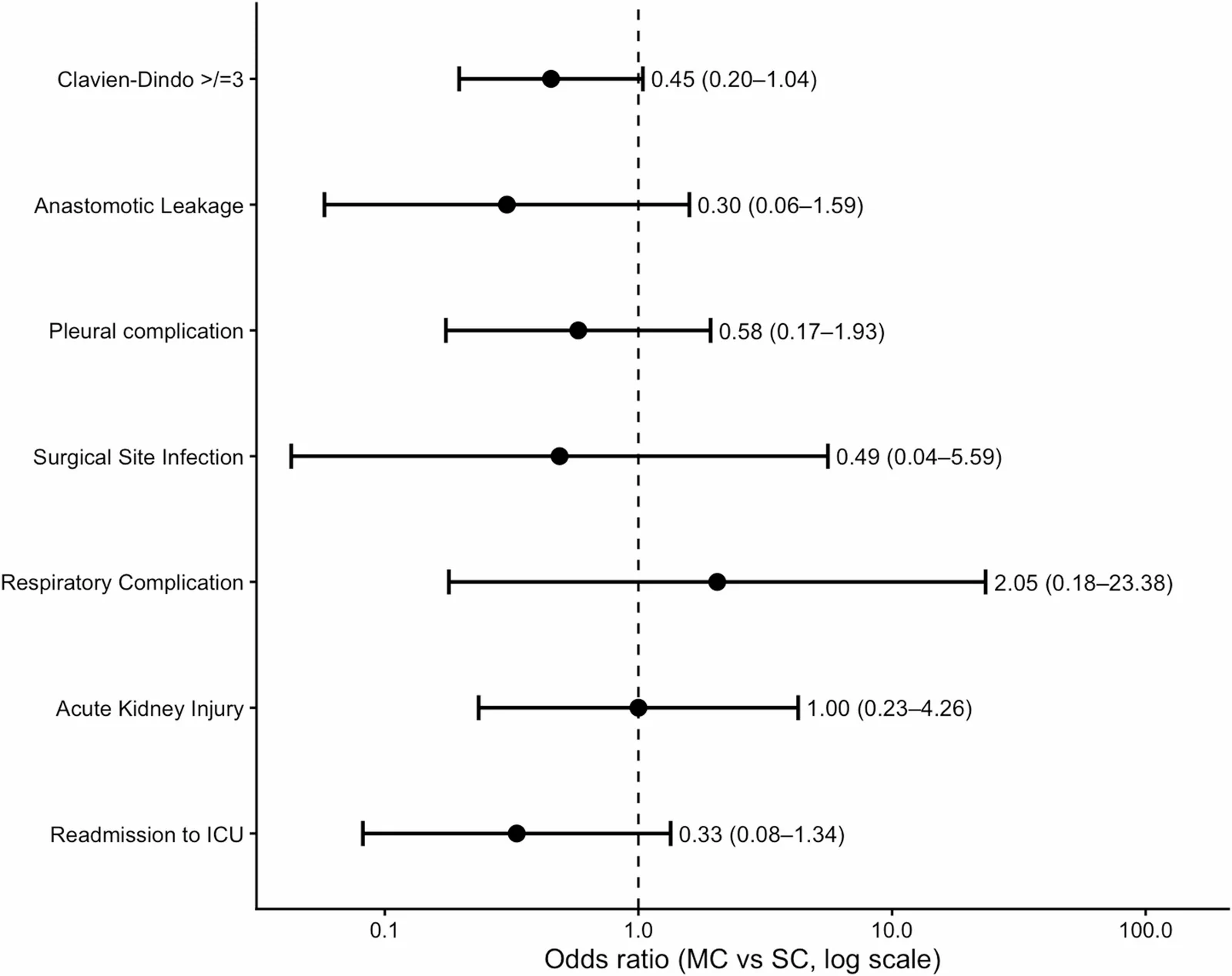
Forest plot showing odds ratios with 95% confidence intervals for postoperative outcomes comparing modified versus standard care groups in the propensity-score matched cohort. Odds ratios <1 indicate lower odds of the outcome in the MC group

Effect estimates for postoperative outcomes after PSM are summarized in a forest plot (Fig. 1). Across most endpoints, odds ratios favored the modified perioperative care regimen, with point estimates consistently below unity for anastomotic leakage, pleural complications, surgical site infection, and IMC readmission. The odds of major complications were lower in the modified-care group (OR 0.45, 95% CI 0.20–1.04), although this did not reach statistical significance. Similarly, anastomotic leakage occurred less frequently in the modified-care group (OR 0.30, 95% CI 0.06–1.59). No statistically significant differences were observed for pleural complications, surgical site infection, respiratory complications, acute kidney injury, or IMC readmission, as reflected by confidence intervals crossing unity. Deep abscesses were too infrequent to allow reliable estimation of an effect size and were therefore not included in the graphical display.

**Fig. 2:**
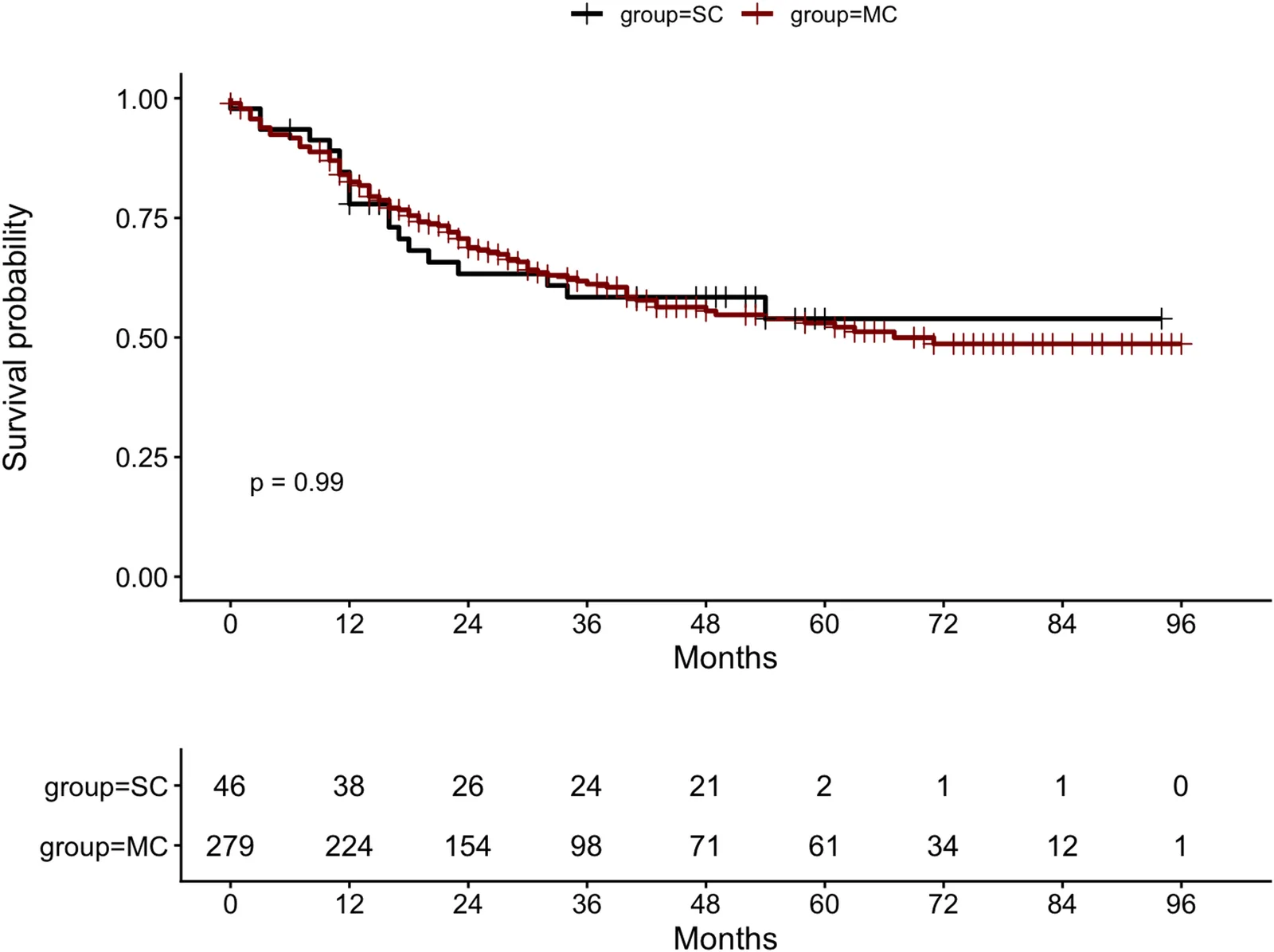
Kaplan-Meier plot showing overall survival. mOS = 71 months in modified care cohort. Log rank (Mantel-Cox) test n. s

**Fig. 3:**
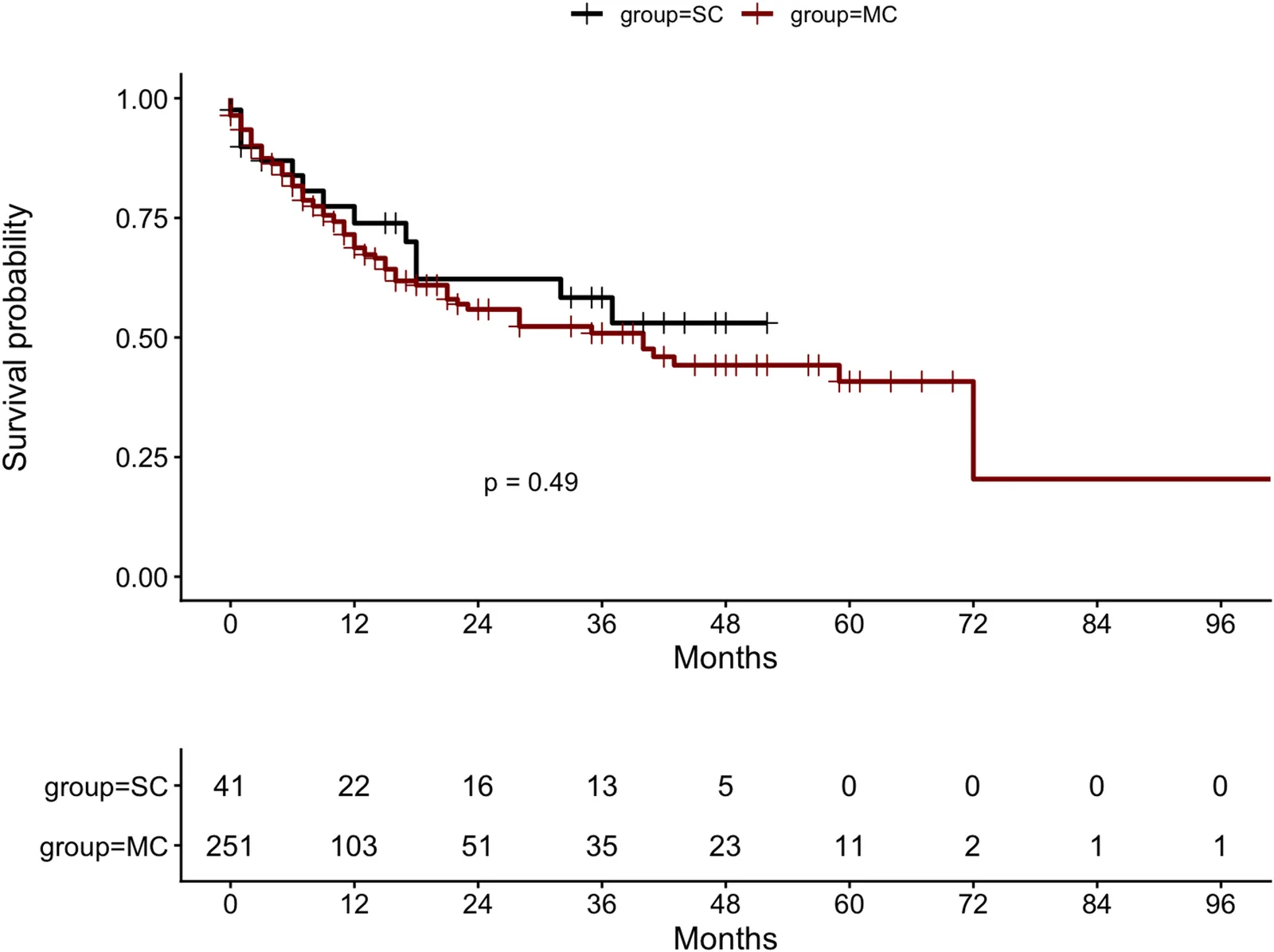
Kaplan-Meier plot showing disease-free survival. mDFS = 40 months in modified care group. Log rank (Mantel-Cox) test n. s

**Fig. 4:**
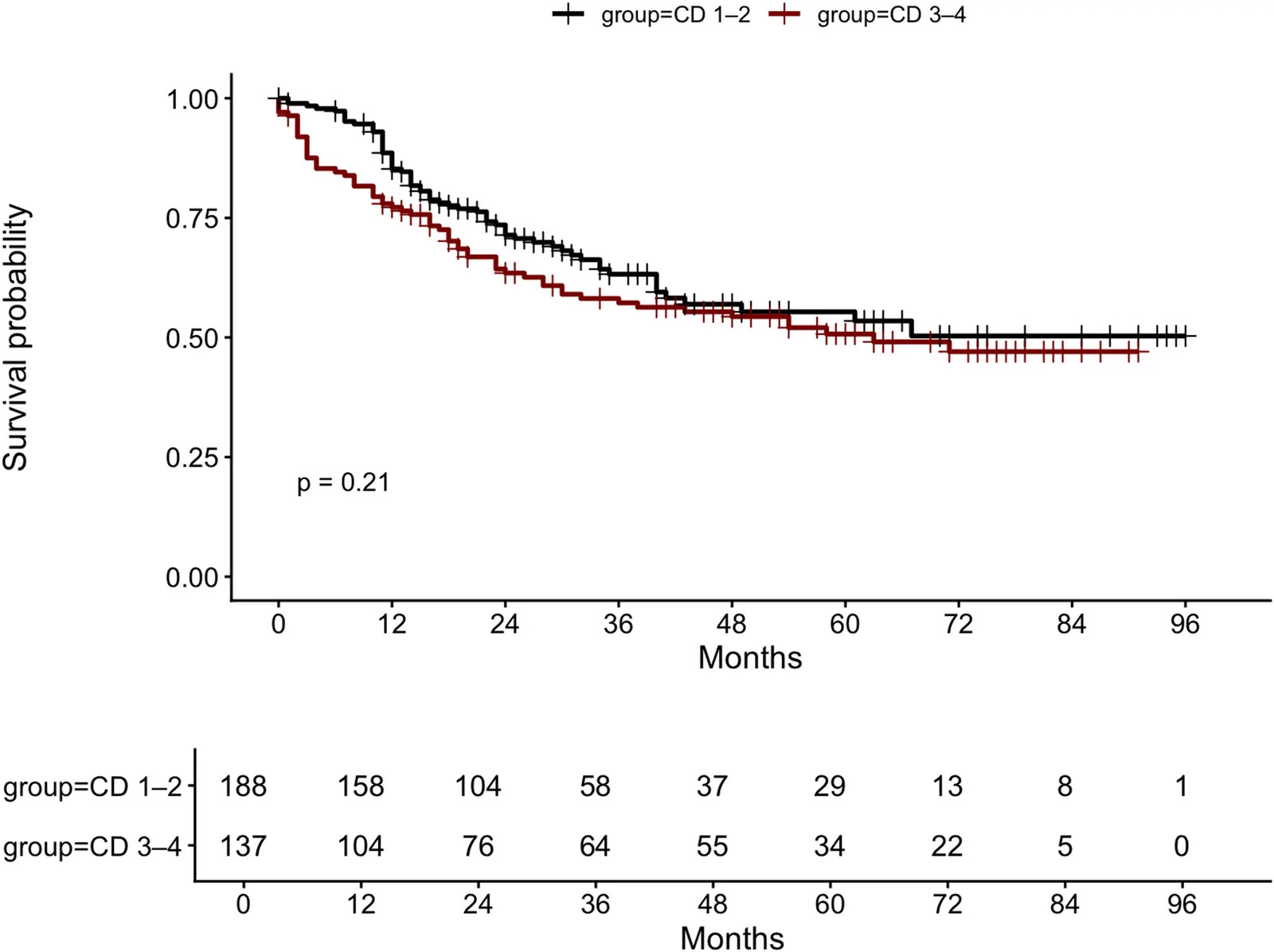
Kaplan-Meier plot showing overall survival for minor vs. major complications. Log rank (Mantel-Cox) test n. s

Overall and disease-free survival did not differ between groups in the unmatched cohort. Median overall survival in the standard-care group was not reached during follow-up, and complication severity was not associated with reduced overall survival (Figs. 2, 3 and 4).

## Discussion

In this retrospective propensity score-matched analysis of patients undergoing two-field esophagectomy, the primary endpoint was postoperative morbidity assessed by the Comprehensive Complication Index (CCI). In the unmatched cohort, mean CCI was lower in patients receiving modified care (28.92, 95% CI 25.60–32.23) than in those receiving standard care (35.70, 95% CI 21.32–51.60). After propensity score matching, the mean CCI in the modified-care group was 32.01 (95% CI 23.56–40.45), with the between-group difference no longer reaching statistical significance.

Among secondary outcomes, complication rates were comparable but anastomotic leakage was less frequent in the modified-care group in the unmatched analysis, although this difference likewise did not remain statistically significant after matching. No increase in acute kidney injury or renal replacement therapy was observed despite the use of a restrictive fluid strategy and HES in the modified pathway.

Among 325 cases, only 20% were squamous cell carcinomas (SCC). Most cases (63%) presented with advanced-stage tumors (cT3 or cT4), and 65% had lymph node involvement at diagnosis. Therefore, a majority (85%) received neoadjuvant treatment in accordance with current guidelines for locally advanced esophageal cancer. The high frequency of locally advanced tumors, lymphatic spread, as well as the rate of neoadjuvant treatment are consistent with published western series and predispose to and relativize the overall high frequency of postoperative complications [4, 39].

While benchmark multicentric studies (e.g. ECCG, DUCA [4, 40]) have demonstrated age, BMI, ASA-score, N+-status and open transthoracic approach to be associated with increased morbidity, advanced disease, high BMI and comorbidities were even more frequent and open transthoracic approach was standard at our center during the observed period, overall outcomes remained comparable with benchmark cohorts.

Evidence for oral decontamination to reduce the risk of developing and severity of pulmonary complications after esophagectomy remains limited [41], although studies suggested potential reductions in pulmonary complications in upper gastrointestinal cancer surgery [16, 17, 19]. In our unmatched cohort, there was no discernible difference between MC and SC in this regard, but pulmonary complications were slightly more frequent in MC cohort after matching, which is also reflected in an OR of 2.05 [0.18–23.38]. The small cohort size after matching coupled with very few absolute events limit interpretability of this result, though, as no statistical significance was reached. The same caution must be applied to the interpretation of odds ratios calculated for binary endpoints anastomotic leak, pleural complication and surgical site infection. While some trends may be observed, none reached statistical significance. While the isolated OR for respiratory complications seems to favor SC, the absolute incidence of single complications in either group was lower than in published benchmark series and the apparent difference must be interpreted with caution. While anastomotic leakages were significantly less frequent in MC in the unmatched cohort, this did not reach statistical significance after PSM. Like other binary endpoints, the overall frequency of events in the small matched cohort for anastomotic leakage limits statistical interpretability. The observed reduction remained as trend just shy of statistical significance.

To achieve fluid balance, loop diuretics were used frequently in both groups (82.6% standard of care, 85.3% modified care) throughout the first 4 postoperative days, this is even more frequent than in many published series [42] and resulted in moderate fluid balances even in SC group, but significantly lower balances in MC group. This effect did not persist significant but remained as trend after PSM. This may partly reflect the routine application of HES solutions and reduced intravenous fluid replacement. Even in the standard of care group, balance on POD 4 remained below published thresholds for increased risk of anastomotic complications [43].

Patient-controlled analgesia was not employed in our cohort, patients routinely received thoracic epidural anesthesia (TEA) and non-opioid analgesia (standard Metamizole 1000 mg 4x/d) in both cohorts with parenteral opioids (mainly transdermal fentanyl) as fallback in TEA failure which was uncommon. The lower fluid balance observed in MC may therefore be related to the protocolized fluid/diuretics strategy and reduced intravenous fluid administration rather than TEA-associated vasoplegia.

Remarkably, the overall frequency of acute kidney injury AKI was very low in our cohort, whereas in a recently published investigation of AKI after esophageal cancer surgery, about 18% of patients suffered from AKI according to the full KDIGO definition including oliguria [44]. We did not retrospectively evaluate urine output and therefore likely underestimated the incidence that would be indentified using the full KDIGO definition. Nonetheless, this is relevant given the use of HES in the modified pathway.

HES solutions have been widely used for intravascular volume expansion in surgical patients, but some concerns have been raised since an influential RCT [29] demonstrated an increased incidence of renal replacement therapy and need for blood transfusion after application of HES in critically ill patients [45, 46]. Our data on the other hand suggest it may still play an important role in perioperative fluid management when used appropriately. This is in line with a multicenter study of patients undergoing major abdominal surgery where HES application resulted in improved hemodynamic stability and reduced need for vasopressors when compared to saline [47]. The enhanced volume effect is thought to be beneficial in maintaining adequate tissue perfusion especially at the critically perfused esophagogastric anastomosis during the immediate postoperative period. While oliguric renal failure is frequent in published series, in our cohort, anuria, significant biochemical renal failure or need for renal replacement therapy were not significantly increased. While several randomized controlled trials have investigated HES application in elective surgery [48], we observed its safety in a large cohort of patients undergoing two-field esophagectomy with a risk profile distinct from major abdominal or orthopedic procedures.

Postoperative nutrition and the optimal timing and route of nutritional support after esophagectomy is a critical aspect of patient recovery. Recent literature suggests early oral feeding to lead to shorter hospital stays and quicker bowel recovery, although it may potentially increase anastomotic leakage [49, 50]. Conversely, late oral feeding with jejunostomy support has shown less body weight loss and better nutritional intake [51], but necessary nasojejunal tube or jejunostomy placement can be associated with complications of its own [52]. Sole parenteral nutritional support has shown similar rehabilitation outcomes [53] and was therefore chosen for nutritional support at our center. The early initiation of parenteral nutrition (PN) was a key distinction between our regimens. Modified group (MC) had a significantly higher frequency of early PN initiation (33% vs. 8.7%, *p* < 0.01), and this persisted after matching (32.6% vs. 8.7%, *p* = 0.04). The significantly longer duration of parenteral nutrition in MC (1.1 days vs. 7.7 days, *p* = 0.044) further emphasizes the early intervention strategy in these patients, although this difference did not remain significant after PSM.

Despite mandatory postoperative gastric decompression in MC, timing of NG tube removal did not differ significantly between groups. Thus, our data do not demonstrate an independent effect of the decompression strategy on this outcome.

This study has several important limitations that must be acknowledged. First, the absence of total minimally invasive esophagectomy (TMIE) procedures limits the generalizability of our findings to current surgical practice. Since the study period, advances in thoracoscopic and robotic techniques have markedly reduced perioperative morbidity, anastomotic complications, and recovery times, thereby redefining the standard of care for esophageal cancer surgery. Second, SC cohort was comparatively small, which may have restricted statistical power to detect smaller but clinically relevant differences between groups. Furthermore, selection and observation biases cannot be excluded as relevant limitations and the long study period introduces the risk of historical bias where increased institutional experience and broader perioperative quality improvement over time may have influenced results. The retrospective, single-center nature of this analysis inherently limits causal inference and may be influenced by unmeasured confounders despite the use of propensity score matching.

Nevertheless, our findings provide meaningful real-world evidence from a high-volume tertiary center with complication frequency and severity below published benchmark series and suggest that selective modifications of perioperative management can be implemented safely and warrant prospective evaluation aimed at refining perioperative care in esophagectomy.

## Conclusion

In this retrospective propensity score-matched analysis of 325 patients undergoing two-field esophagectomy, a modified postoperative care pathway differing from ERAS^®^ recommendations was evaluated with postoperative morbidity assessed by the Comprehensive Complication Index as the primary endpoint. The unmatched analysis showed lower overall morbidity and fewer anastomotic leakages in the modified-care group, these differences remained as trends shy of statistical significance after propensity score matching. No increase in clinically relevant renal complications or renal replacement therapy was observed despite the use of a restrictive fluid strategy and HES. These findings suggest that the modified pathway can be implemented safely in this institutional setting. Given the retrospective design, its limitations, and overall low frequency of complications, prospective studies are warranted to determine whether individual components of the modified pathway can improve perioperative outcomes after esophagectomy.

## Data Availability

The data generated and analyzed during the current study are not publicly available due to German data protection regulations and restrictions imposed by the patient consent forms, which do not permit unrestricted data sharing. De-identified data may be made available from the corresponding author upon reasonable request and subject to approval by the institutional ethics committee and applicable data protection authorities.
